# Supplementary material for: Inactivation of mrpigH Gene in Monascus ruber M7 Results in Increased Monascus Pigments and Decreased Citrinin with mrpyrG Selection Marker
Source: J Fungi (Basel). 2021 Dec 19;7(12):1094. doi: 10.3390/jof7121094 (PMC8705778; doi:10.3390/jof7121094)
Supplement: Supplementary file 1 [file jof-07-01094-s001.zip › jof-1491459-supplementary.pdf]

Table S1. Primers used in RT-qPCR

Names	Sequences (5′⟶ 3′)	Descriptions
mrpigA qF	CTCGAGGAATTGAGCGTTGG	For RT-qPCR analysis of *mrpigA*
mrpigA qR	CAGGAAGACTCAATTCGCCG	(187 bp)
mrpigB qF	CAGAAACCATCACGCAGGAG	For RT-qPCR analysis of *mrpigB*
mrpigB qR	AAAGAAGCAGCGGGTCTACT	(246 bp)
mrpigC qF	CCTACCCAGCAATCGATCCT	For RT-qPCR analysis of *mrpigC*
mrpigC qR	ACGTCCTTTGCTAGCTCTGT	(169 bp)
mrpigD qF	GTACGCGGGGAAGTTCAATC	For RT-qPCR analysis of *mrpigD*
mrpigD qR	CCCCAATATCCTCCCTCGTC	(212 bp)
mrpigE qF	CTGTACAACGTCCTGCATCG	For RT-qPCR analysis of *mrpigE*
mrpigE qR	TCTCCCGAATCGTATCCAGC	(247 bp)
mrpigF qF	GTCACGTCTCAGATCGCAAC	For RT-qPCR analysis of *mrpigF*
mrpigF qR	CTGGCACTGTCGATGAACTG	(209 bp)
mrpigG qF	TACAAGGAGTTCGGGCCATT	For RT-qPCR analysis of *mrpigG*
mrpigG qR	GCAGGCTAGCACACATCTTC	(199 bp)
mrpigH qF	TCGTCTCGTGGATCATCTCG	For RT-qPCR analysis of *mrpigH*
mrpigH qR	GATGCTCTCCAATCCCTTGC	(197 bp)
mrpigI qF	CATCTTGGACGGGATTGCAG	For RT-qPCR analysis of *mrpigI*
mrpigI qR	ATCTCGTCCTTGCTCACACA	(162 bp)
mrpigJ qF	CGTTTCGGCTGATCATTCGT	For RT-qPCR analysis of *mrpigJ*
mrpigJ qR	CGATCCCGCTGAAGAACTTG	(236 bp)
mrpigK qF	CAATCGGACGGAAATGACC	For RT-qPCR analysis of *mrpigK*
mrpigK qR	CTTTGAGTCTCATCGCCAGC	(176 bp)
mrpigL qF	TCAGGGATTGTGGGATTGCT	For RT-qPCR analysis of *mrpigL*
mrpigL qR	CTTGCATCGCCTTGTCAACT	(220 bp)
mrpigM qF	GTGACTTTGAACAGCCTGGG	For RT-qPCR analysis of *mrpigM*
mrpigM qR	CGCTCAATTCCTTCTCCAGC	(241 bp)
mrpigN qF	CGATGCAATGGGGAGAGAGA	For RT-qPCR analysis of *mrpigN*
mrpigN qR	CGAATCCAGAGAAGGCTTGC	(200 bp)
mrpigO qF	AACTGCTCTTCGAGACGGAT	For RT-qPCR analysis of *mrpigO*
mrpigO qR	CGAACTCCAGCAGCAACTTC	(168 bp)
mrpigP qF	CTATTTGGTGCGGACGAGTG	For RT-qPCR analysis of *mrpigP*
mrpigP qR	TCCAACACCTCTTCGATGCT	(186 bp)
Beta-actin F	TCTGGCACCACACATTCTACAA	For RT-qPCR analysis of *beta-*
Beta-actin R	CGAAGACGATCTGGGTCATCT	*actin* (120 bp)
mrl7 qF	GAAGTGATTCTCAGCGCTGG	For RT-qPCR analysis of *mrl7*
mrl7 qR	AGAGCGGGATCCTTGAACAA	(215 bp)
mrl6 qF	GCCATGCTGCCTCTTCTTTT	For RT-qPCR analysis of *mrl6*
mrl6 qR	ACTTTGCCTTGGTGTCTTCG	(241 bp)
mrl5 qF	ATGTCTGCTATCCCTCCTGC	For RT-qPCR analysis of *mrl5*
mrl5 qR	TAGAGGTAGAGCTTGGTGGC	(242 bp)

Table S1. Primers used in RT-qPCR

Names	Sequences (5′→ 3′)	Descriptions
mrl4 qF	CGCTTGTCAAGATGGTCTCG	For RT-qPCR analysis of *mrl4*
mrl4 qR	TCGCCCTTGTTGAAGAAAGC	(190 bp)
mrl3 qF	CCGAGCCAGACATGTCATTG	For RT-qPCR analysis of *mrl3*
mrl3 qR	GATGTCTTCTTCGCGGCATT	(217 bp)
mrl2 qF	AGACATTCCCCTCGACGATC	For RT-qPCR analysis of *mrl2*
mrl2 qR	GCGACAAGGTCCAACACTAC	(165 bp)
mrl1 qF	GTCTGATGCTGAGCCCAATG	For RT-qPCR analysis of *mrl1*
mrl1 qR	CGGATTTGTTGGCGGTAGAG	(246 bp)
mrpks qF	AAGCCAATATTCAGCGCCTG	For RT-qPCR analysis of *mrpks*
mrpks qR	GCACCAGTAACAAGCACACA	(235 bp)
mrr1 qF	GTCGTACCGGAAGATCGTCT	For RT-qPCR analysis of *mrr1*
mrr1 qR	TAGTTCAGGCCTTGCATCCA	(173 bp)
mrr2 qF	ATGATCTGGAAGGCTACGGG	For RT-qPCR analysis of *mrr2*
mrr2 qR	GGCACCTTGGTCTTCTCTCT	(196 bp)
mrr3 qF	CAGTCCAAGAACCGCTATGC	For RT-qPCR analysis of *mrr3*
mrr3 qR	AAACTGGGATGGGAGGATGG	(171 bp)
mrr4 qF	GGGCACACATCCATCGATTC	For RT-qPCR analysis of *mrr4*
mrr4 qR	GCGTCGTAGTTAAAGGCTCG	(239 bp)
mrr5 qF	ACATCCTTCACCCAACCGAT	For RT-qPCR analysis of *mrr5*
mrr5 qR	TCAGTTCGGTCAGCTTCAGT	(246 bp)
mrr6 qF	CTTTCCTACTCCTCCGCCAA	For RT-qPCR analysis of *mrr6*
mrr6 qR	CGAAGAAAGCGATCAGGACG	(160 bp)
mrr7 qF	TCGAGAAGACAGAACGTGCT	For RT-qPCR analysis of *mrr7*
mrr7 qR	CACAAAGTCAATGCCACCCA	(152 bp)
mrr8 qF	CCCAAACCCGCTATTGTCAG	For RT-qPCR analysis of *mrr8*
mrr8 qR	CCGACCGTTAAACAAGCACA	(158 bp)
